# Non-Canonical Wnt11 Signaling Regulates Pulmonary Fibrosis via Fibroblast and Alveolar Epithelial Type II Cell Crosstalk

**DOI:** 10.3390/ijms27010351

**Published:** 2025-12-29

**Authors:** Francina Gonzalez De Los Santos, Akira Ando, Biao Hu, Alyssa Rosek, Sem H. Phan, Tianju Liu

**Affiliations:** 1 Graduate Program in Immunology, University of Michigan, Ann Arbor, MI 48109, USA; gonzalef@umich.edu; 2 Department of Respiratory Medicine, Nagoya University Graduate School of Medicine, Showa-ku, Nagoya 466-8550, Japan; 3 Department of Pathology, University of Michigan Medical School, Ann Arbor, MI 48109, USA

**Keywords:** Wnt11, fibrosis, epithelial cell and fibroblast crosstalk

## Abstract

The reactivation of Wnt signaling pathways plays an important role in driving myofibroblast differentiation in fibrotic diseases; however, the mechanism is not clearly understood. In this study, we investigate the role of non-canonical Wnt11 signaling in human lung fibroblasts and its contributions to myofibroblast differentiation. Our results show that components of the non-canonical Wnt pathway are upregulated in bleomycin-induced pulmonary fibrosis and that in vivo depletion of Wnt11 in mouse lung fibroblasts significantly reduces lung fibrosis. Furthermore, co-culture studies using fibroblasts and alveolar type II epithelial cells (AECII) revealed a Wnt11-mediated mechanism that promotes myofibroblast differentiation. Finally, we demonstrate that in human lung fibroblasts, TGFβ can increases Wnt11 transcription by regulating Smad3 binding to the Wnt11 promoter and by modulating Wnt11 promoter activity. Together, these findings identify non-canonical Wnt11 as a regulator of myofibroblast differentiation and lung fibrosis.

## 1. Introduction

Reactivation of Wnt signaling pathways plays an important role in regulating many aspects of development, from stem cells to fully differentiated cells, tissues and organs, including myofibroblast genesis and differentiation, a key component of lung repair and remodeling [1,2]. Wnt signaling can be divided into canonical and non-canonical pathways, depending on whether the pathway operates with or without β-catenin, respectively [3,4,5]. Despite the ample evidence showing reactivation of Wnt signaling pathways in fibrotic diseases, the specific roles of individual Wnt ligands and their diverse target genes, cellular processes, and affected cell types remain poorly defined. A growing body of evidence suggests that non-canonical ligands, including Wnt5a, Wnt7a and Wnt11, contribute to fibrosis across multiple organs such as the lung, kidney and heart [6,7,8,9,10,11,12]. Wnt11 is upregulated in both kidney and pulmonary fibrosis, and it has been shown to promote myofibroblast and cardiomyogenic differentiation through c-Jun/JNK pathway, acting in both autocrine and paracrine manners [6,8,11].

In our previous work, we had shown that both canonical Wnt3a/β-catenin and non-canonical Wnt11 signaling are involved in the differentiation of human lung fibroblasts into myofibroblasts. Wnt3a/β-catenin can activate non-canonical Wnt11 via β-catenin signaling, while non-canonical Wnt11-induced myofibroblast differentiation occurs through the JNK/c-Jun pathway independently of Wnt3a/β-catenin [6]. The canonical and non-canonical Wnt signaling pathways are interconnected; our studies and others have shown that the canonical pathway can activate the non-canonical pathway, which in turn inhibits the canonical pathway via a negative feedback mechanism [6,13,14]. We speculate that Wnt11 may also play a paracrine role in modulating myofibroblast differentiation during fibrosis by mediating the crosstalk between epithelial cells and fibroblasts [6,14].

The specific role of Wnt11 in fibroblasts during pulmonary fibrosis, as well as its function in cellular crosstalk, remains unclear. In this study, we investigated the impact of Wnt11 signaling in fibroblasts and pulmonary fibrosis in vivo using a fibroblast-specific Wnt11 knockout model. We also explored Wnt11-mediated crosstalk between alveolar type II epithelial cells (AECIIs) and fibroblasts, as well as the transcriptional regulation of Wnt11 in fibroblasts.

## 2. Results

### 2.1. Wnt11 Induction Is Dependent on β-Catenin in Human Lung Fibroblasts (HLF)

**Figure 1:**
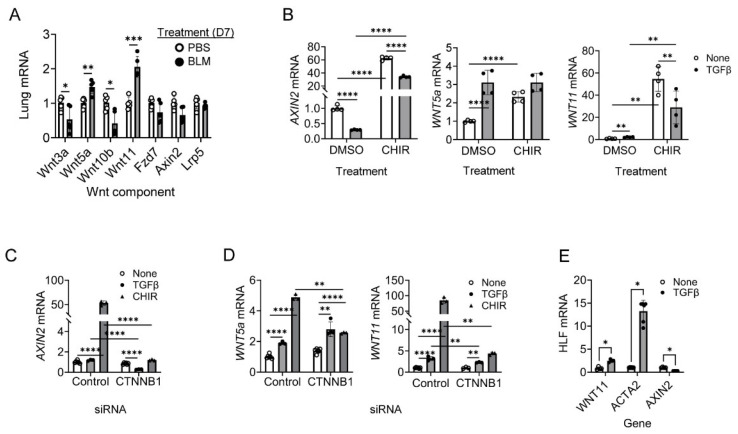
Wnt11 induction in human lung fibroblast is β-catenin dependent. (**A**) Lung tissue RNA from PBS- or BLM-treated mice at day 7 were analyzed for Wnt component mRNA *N* = 5. (**B**) Human lung fibroblasts (HLFs) were pretreated with GSKβ inhibitor CHIR (5 µM) or solvent DMSO (None) for 24 h, followed by treatment with TGFβ (5 ng/mL) or PBS (None) for 24 h. The cells were harvested after the indicated hours and analyzed for their mRNA level of AXIN2, WNT5a and WNT11 *N* = 3–6. HLFs were transfected with control or β-catenin (CTNNB1) siRNA (50 nM) for 24 h and then treated with TGFβ (5 ng/mL) or CHIR99021 (CHIR, 5 µM) for 48 h. Total RNA from HLF was analyzed by qPCR to determine the mRNA levels of AXIN2 (**C**), WNT5A and WNT11 (**D**). (**E**) HLFs were treated with TGFβ (5 ng/mL) or PBS (None) for 24 h and total RNA was analyzed by qPCR to determine the mRNA levels of WNT11, ACTA2 and AXIN2. Numbers above the bars indicate fold-change from untreated cells. *N* = 3–6. *p* < * 0.05, ** 0.01, *** 0.001, **** 0.0001 between the indicated two groups by one-way ANOVA plus Tukey’s or Student’s *t*-test.

Given the differential mechanisms through which canonical and non-canonical Wnt signaling pathways contribute to lung myofibroblast differentiation [6], we examined the expression patterns of Wnt components in mouse lungs 7 days after BLM installation. This time point is characterized by inflammation and, interestingly, the expression of canonical Wnt components, including Wnt3a and Wnt10b, was significantly reduced in the injured lung at day 7. Other components of the Wnt signaling cascade, such as Fzd7 and LRp5, remained unchanged after bleomycin treatment. In contrast, non-canonical Wnt11 and Wnt5a gene expression was significantly upregulated at day 7 post-bleomycin treatment (Figure 1A). These data demonstrate that canonical and non-canonical Wnt signaling are differently regulated in bleomycin-induced fibrosis in mice.

To examine the role of canonical Wnt signaling in myofibroblast differentiation, we used the GSK3β inhibitor CHIR99021 as an activator of canonical Wnt3a/β-catenin signaling. Treatment of human lung fibroblasts (HLFs) with CHIR99021 increased Axin2 expression, a direct target of canonical Wnt3a/β-catenin signaling, as expected (Figure 1B). The non-canonical Wnt ligands Wnt5a and Wnt11 were also significantly induced by CHIR99021, with Wnt11 mRNA in particular increasing by nearly 60-fold (Figure 1B), confirming the previous finding that canonical Wnt3a/β-catenin signaling induces non-canonical Wnt11 signaling during myofibroblast differentiation [6]. Additionally, CHIR99021-induced Wnt11 expression was suppressed by TGFβ, indicating that TGFβ can partially inhibit canonical Wnt activation in HLFs. TGFβ treatment alone modestly increased Wnt11 gene expression, but not to the same extent as CHIR99021 (Figure 1B). These data indicate that, in fibroblasts, the canonical Wnt pathway plays a more dominant role than TGFβ in the regulation of Wnt11 expression.

It is well established that β-catenin, as a key effector of canonical Wnt signaling, is required for Wnt3a-induction of myofibroblast differentiation, acting indirectly through Wnt11, and that β-catenin is also upregulated/activated by CHIR99021 [6,15]. To further clarify β-catenin dependence of CHIR99021- mediated activation of canonical Wnt signaling and myofibroblast differentiation, β-catenin was knocked down with siRNA prior to TGFβ or CHIR99021 treatment in HLFs. β-catenin knockdown abrogated CHIR9902-induced AXIN2 mRNA expression, confirming β-catenin dependence in the canonical Wnt pathway (Figure 1C). Notably, β-catenin knockdown also eliminated CHIR99021-induced expression of the non-canonical ligands Wnt5a and Wnt11, with a more pronounced effect on Wnt11 (Figure 1D). To assess how TGFβ influences the balance between canonical and non-canonical Wnt pathways, HLFs were treated with TGFβ for 24 h. TGFβ upregulated the fibrotic marker ACTA2 and non-canonical ligand WNT11, while downregulating of the canonical target AXIN2 (Figure 1E). Together, these findings indicate that WNT11 induction in lung fibroblasts is regulated by both β-catenin and TGFβ signaling.

### 2.2. Mesenchymal Cell-Specific Wnt11 Deficiency In Vivo Impairs Pulmonary Fibrosis and Myofibroblast Differentiation

**Figure 2:**
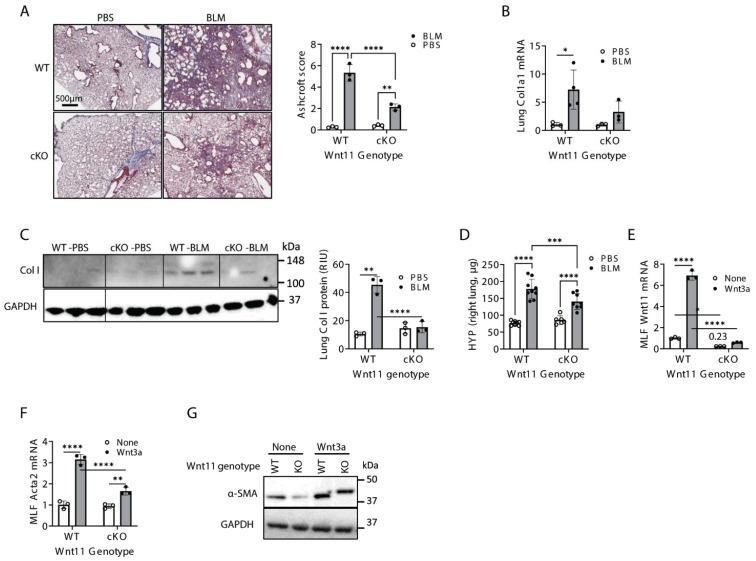
Mesenchymal-specific Wnt11 deficiency impaired pulmonary fibrosis and myofibroblast differentiation. Lung fibrosis was induced by bleomycin (BLM) administration following 7 days of tamoxifen injections. (**A**) Representative Trichrome-stained lung tissue sections are shown (scale bar: 500 μm), and Trichome staining was quantified using the Ashcroft scoring method. (**B**) Lung collagen I expression was analyzed at mRNA and protein levels by qPCR (**B**) and Western blotting with quantification (**C**). (**D**) Lung tissue from WT or Wnt11^fl/fl^-ColCre^+/−^ cKO mice (cKO) were homogenized at day 21 after BLM treatment and analyzed for collagen content measured by hydroxyproline assay. Mouse lung fibroblasts (MLFs) isolated from BLM-treated WT or cKO mice were further treated with Wnt3a (200 ng/mL) in vitro. MLF RNA collected at 24 h was analyzed for mRNA expression of Wnt11 (**E**) and a-SMA (Acta2) (**F**). MLF lysate collected at 48 h was analyzed for a-SMA protein expression by Western blotting. A representative blot is shown (**G**). *N* = 3–5. *p* < * 0.05, ** 0.01, *** 0.001, **** 0.0001 by one-way ANOVA plus Tukey’s or Student’s *t*-test.

Our previous work suggested that Wnt3a/β-catenin promotes myofibroblast differentiation in an autocrine fashion [6]. To further investigate the role of Wnt11 in vivo, we generated conditional, tamoxifen-inducible, mesenchymal collagen I-expressing cell-specific Wnt11 knockout mice (Wnt11^fl/fl^-ColCre^+/−^ conditional knockout; referred to as cKO mice). Mice were treated with bleomycin for 21 days, after which whole lungs were collected for analysis. Histopathological assessment revealed that cKO lungs exhibited markedly less fibrosis and reduced collagen deposition compared to the WT controls, as quantified using the Ashcroft scoring method (Figure 2A). Consistent with these findings, Col1a1 mRNA and protein levels were decreased in bleomycin-treated cKO lungs (Figure 2B,C), and bleomycin-induced lung hydroxyproline content was significantly reduced in cKO mice (Figure 2D). These results are notable because fibroblast-specific Wnt11 depletion produced a substantial reduction in collagen I expression, a key driver of fibrosis, indicating that fibroblasts are a major source of Wnt11 in vivo.

To further examine fibroblast-intrinsic effects, collagen I-expressing mouse lung fibroblasts (MLFs) were isolated from cKO and wild-type mice. Wnt11 expression was significantly reduced in cKO MLFs compared to WT MLFs (1 vs. 0.23). Moreover, Wnt11-deficient MLFs failed to respond to canonical Wnt3a stimulation, whereas Wnt3a stimulation caused a more than 6-fold increase in Wnt11 mRNA in WT MLFs (Figure 2E). This reduction in Wnt11 also significantly blunted α-SMA mRNA (Acta2) induction, with WT MLFs exhibiting a 3.1-fold increase compared to only 1.7-fold in cKO MLFs (Figure 2F). A similar pattern was observed at the α-SMA protein level (Figure 2G). Together, these findings indicate a protective effect of Wnt11 deficiency in the mesenchymal compartment in vivo. Loss of Wnt11 in fibroblasts markedly reduces myofibroblast differentiation and attenuates fibrosis.

### 2.3. Non-Canonical Wnt11 Promotes Myofibroblast Differentiation via a Paracrine Mechanism>

**Figure 3:**
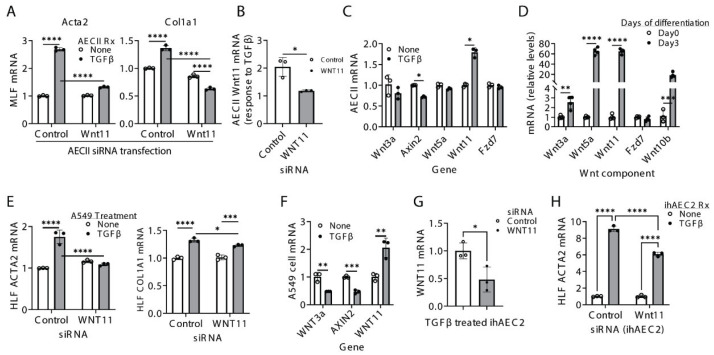
AECII-derived Wnt11 promotes lung myofibroblast differentiation via paracrine signals. (**A**) Primarily isolated mouse AECIIs, A549 cells or ihAEC2s were transfected with the indicated siRNA for 24 h and then treated with TGFβ for another 48 h. After replacing the media, they were co-cultured in Transwell plates with mouse lung fibroblasts (MLFs) or HLF for 48 h. MLFs were harvested and analyzed for mRNA expression of α-SMA (Acta2) and collagen I (Col1a1) by qPCR (**A**). (**B**) AECII RNA samples from (**A**) were analyzed for Wnt11 mRNA by qPCR. The *y*-axis presents the fold-changes of TGFβ-induced Wnt11 from their untreated samples. (**C**) AECII RNA samples from (**A**) were analyzed for the indicated mRNA by qPCR (fold-change from respective untreated control). (**D**) Primary AECII cultured in 2D on a stiff substrate coated with laminin were collected on day 0 and 3 and analyzed for the indicated mRNA Wnt components by qPCR. (**E**) A549 cells were transfected with WNT11 siRNA and then treated with TGFβ as in (**A**), followed by co-culturing with HLFs for 48 h. HLFs were harvested and analyzed for ACTA2 and COL1A1 mRNA by qPCR. (**F**) RNA samples were isolated from A549 cells after TGFβ or PBS treatment and analyzed by qPCR for WNT3a, AXIN2 and WNT11 gene expression (fold-change from respective untreated control). (**G**) WNT11 mRNA expression was measured by qPCR in iHAEC2s after siRNA knockdown and TGFβ treatment. (**H**) Human lung fibroblast ACTA2 mRNA was measured following co-culture with control or Wnt11-siRNA-treated ihAEC2s with or without TGFβ. *N* = 3–4 in (**A**–**F**). *p* < * 0.05, ** 0.01, *** 0.001, **** 0.0001 between the indicated two groups by one-way ANOVA plus Tukey’s or Student’s *t*-test.

We previously reported that mouse lung alveolar type II epithelial cells (AECIIs) express Wnt11 [6]. We hypothesize that AECII-derived Wnt11 may engage in paracrine crosstalk to activate neighboring fibroblasts. To test this, primary mouse AECIIs were transfected with Wnt11 siRNA and subsequently stimulated with TGFβ before being co-cultured with MLFs in Transwell plates. Fibroblasts co-cultured with TGFβ-stimulated AECIIs showed significant increase in α-SMA and collagen I mRNA. However, these inductions were essentially abolished when AECIIs were pre-transfected with Wnt11 siRNA (Figure 3A). Notably, Wnt11 knockdown also reduced the AECII response to TGFβ by approximately 50% (Figure 3B). Among the Wnt pathway genes examined, Wnt11 was the most strongly induced by TGFβ in control AECIIs, whereas the canonical target gene Axin2 was downregulated (Figure 3C).

In a 2D culture model of alveolar type II epithelial to alveolar type I epithelial cells transitional differentiation [16], non-canonical Wnt11 and Wnt5a were also the most upregulated genes among those analyzed (Figure 3D), suggesting that non-canonical Wnt contributes to epithelial differentiation processes associated with fibrosis. Similar findings were observed in co-culture experiments using HLFs and the human alveolar epithelial A549 cell line. Knocking down Wnt11 in TGFβ-stimulated A549 cells significantly reduced α-SMA and Col1a1 expression in co-cultured HLFs (Figure 3E). Consistently, TGFβ induced a more than two-fold increase in Wnt11, but decreased Wnt3a and Axin2, in A549 cells (Figure 3F).

To further validate these observations in human alveolar type II alveolar epithelial cells, we employed induced human alveolar type II epithelial cells (ihAEC2s), which were derived in vitro from human pluripotent stem cells [17]. At baseline, ihAEC2s expressed no to very low levels of Wnt11, which increased upon TGFβ stimulation. Wnt11 siRNA effectively knocked down TGFβ-induced WNT11 in ihAEC2s (Figure 3G). HLF co-cultured with Wnt11-deficient ihAEC2s showed a significant decrease in ACTA2 gene expression compared with controls (Figure 3H). Together, these findings suggest that AECII-derived Wnt11 promotes fibroblast activation and differentiation through paracrine signaling in the context of fibrosis and that TGFβ acts as a key regulator of WNT11 expression in AECIIs.

### 2.4. Regulation of Wnt11 Transcription and Expression

**Figure 4:**
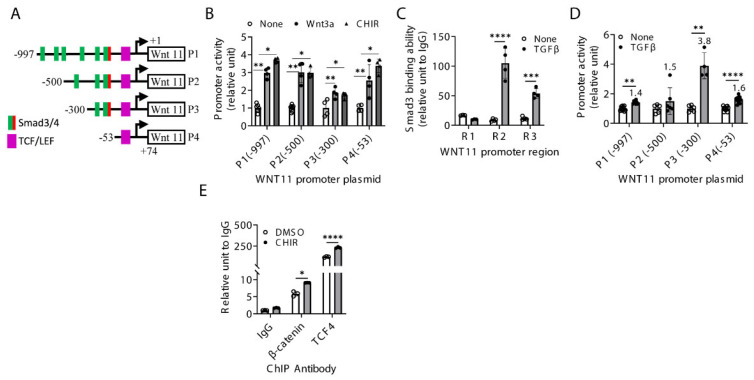
Regulation of Wnt11 transcription and expression. (**A**) Four serial truncated human WNT11 promoter plasmid constructs (P1–P4) are illustrated. The purple box indicates the TCF/LEF binding element, while the green boxes are locations of putative Smad binding elements, with the red box also indicating a single CAGA sequence. (**B**) Four WNT11 promoter constructs were transfected into primary HLFs, respectively, and treated with Wnt3a (200 ng/mL) or CHIR 99021 (CHIR, 5 µM) for 24 h. WNT11 promoter activity was determined after normalization to respective Renilla luciferase activity. (**C**) HLFs were treated with TGFβ for 24 h and analyzed by ChIP assay for the binding of Smad3 to the WNT11 promoter using the primer sets bracketing R1, R2 and R3 regions. The results were expressed as fold-change from their own IgG controls. (**D**) HLFs transfected with each WNT11 promoter were treated with TGFβ (5 ng/mL) for 6 h; then, the activity was examined as described in (**B**). (**E**) CHIR- (5 µM) or DMSO-treated HLF lysates were analyzed by ChIP assay for β-catenin or TCF4 binding to the α-SMA promoter region as in (**B**). *N* = 3–4. Numbers above the bars indicate fold-change from unstimulated cells. *N* = 4–7. *p* < * 0.05, ** 0.01, *** 0.001, **** 0.0001 by one-way ANOVA with Tukey’s or Student’s *t*-test.

Next, we sought to investigate the mechanisms by which TGFβ and canonical Wnt signaling regulate WNT11 transcription and expression. Sequence analysis of the human WNT11 promoter revealed a TCF4/LEF1 binding element located 46 bp upstream of the transcription start site (TTS; +1). In addition, three regions containing multiple putative Smad binding elements (CAGA sequences) were identified at −882 to −827 (Region 1 or R1), −601 to −459 (R2) and −385 to 262 (R3) relative to the TSS site (Figure 4A).

To determine whether canonical Wnt signaling directly activates the WNT11 promoter, four constructs were examined following stimulation with Wnt3a or CHIR99021. All four constructs showed significant basal promoter activity, which was further increased by Wnt3a or CHIR99021 treatment (Figure 4B), confirming the direct transcriptional activation of WNT11 by canonical Wnt signaling through TCF4/LEF complexes.

Next, chromatin immunoprecipitation (ChIP) assays using anti-phospho-Smad3 (pSmad3) antibodies and primer sets spanning regions (R1-R3) were performed in HLFs. Significant pSmad3 binding was detected in all three regions compared with IgG controls. TGFβ treatment enhanced pSmad3 binding across these regions, with the strongest induction observed at R2 and a weaker effect at R3 (Figure 4C).

To assess the functional relevance of these binding sites, the four WNT11 promoter constructs were tested for responsiveness to TGFβ. TGFβ significantly increased WNT11 promoter activity, with greatest induction (3.8-fold) observed for promoter construct P3 (Figure 4D), consistent with enhanced pSmad3 binding. The TGFβ-induced activation of the WNT11 promoter was transient and was no longer evident at 12 or 24 h after treatment.

We next examined whether canonical Wnt signaling directly regulates α-SMA expression in HLFs. Because Wnt3a/β-catenin activates non-canonical Wnt11 and promotes α-SMA induction, we analyzed the human α-SMA promoter for β-catenin/TCF4 binding sites. Sequence analysis identified a putative TCF/LEF consensus binding element (CCTTTGAA) located at −806 relative to the TSS, immediately downstream of a Smad3 binding site at −815. ChIP assays confirmed direct binding of β-catenin and its transcriptional partner TCF4 to the α-SMA promoter, in addition to the known pSmad3 interaction. Notably, CHIR99021 treatment significantly enhanced β-catenin (~2-fold) and TCF4 (~3-fold) binding to the α-SMA promoter (Figure 4E).

These results suggest that WNT11 expression is transcriptionally upregulated by both TGFβ and canonical Wnt signaling in human lung fibroblasts and that WNT11, in turn, contributes to myofibroblast differentiation.

## 3. Discussion

**Figure 5:**
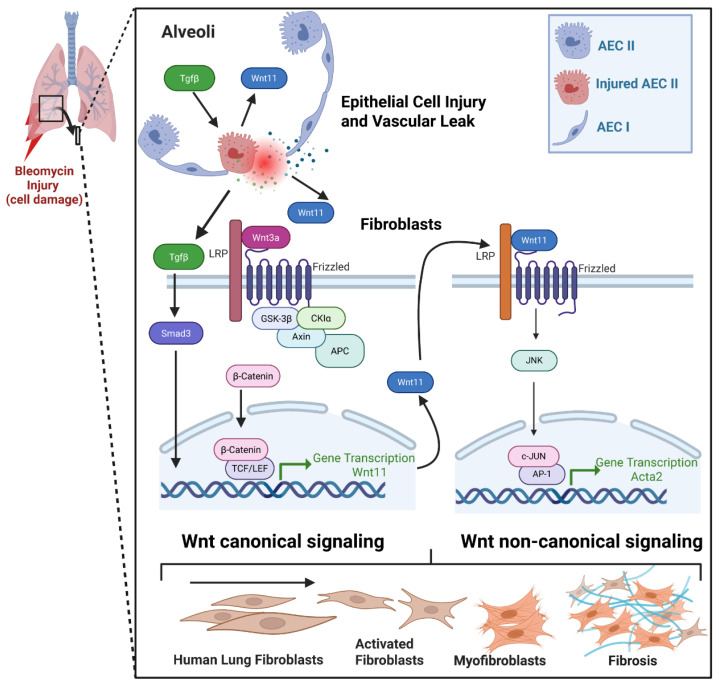
Wnt signaling regulates myofibroblast differentiation. Canonical Wnt signaling is activated when Wnt3a binds to the LRP and Frizzled receptors, triggering an intracellular signaling cascade. Assembly of the destruction complex, comprising GSK-3β, CKIα, Axin and APC, promotes β-catenin phosphorylation and degradation; however, Wnt activation inhibits this process, allowing β-catenin to accumulate and translocate into the nucleus. Nuclear β-catenin then associates with TCF/LEF transcription factors to induce expression of target genes such as Wnt11. Wnt11 can be secreted by fibroblasts and activate non-canonical Wnt signaling by binding to Frizzled receptors and the LRP complex, leading to activation of the JNK/c-Jun pathway and subsequent transcription of Acta2. During bleomycin-induced lung injury, increased TGF-β further stimulates alveolar type II epithelial cells to secrete Wnt11, thereby enhancing paracrine activation of the non-canonical pathway. In addition, TGF-β upregulates Wnt11 transcription through Smad3-dependent mechanisms. Together, canonical and non-canonical Wnt signaling coordinate fibroblast-to-myofibroblast differentiation through distinct yet complementary pathways. Figure generated with BioRender. Created in BioRender. Gonzalez De Los Santos, F. (2025). https://BioRender.com/dvqk9o9.

The Wnt signaling pathway regulates many aspects of development, from stem cells to fully differentiated cells, tissues and organs. Canonical and non-canonical Wnt signaling pathways play distinct roles in embryonic development and tissue homeostasis, influencing cell fate, proliferation and migration [3,4,5,18]. Wnt signaling is also implicated in multiple fibrotic processes, with both canonical and non-canonical pathways contributing to myofibroblast differentiation, a key event in tissue remodeling. However, the precise mechanisms and potential interaction between these pathways remain unclear. In this study, we investigated the role of Wnt11 in a mouse lung fibroblast in the context of fibrosis. We demonstrate that Wnt11 promotes bleomycin-induced lung fibrosis and that fibroblast-specific depletion of Wnt11 significantly reduces fibrotic remodeling. We also reveal novel regulation of Wnt11 expression, potentially through the Wnt3a/β-catenin and TGFβ/Smad3 pathways. Furthermore, we show that Wnt11 released from alveolar type II epithelial cells contributes to myofibroblast differentiation. These findings provide new insights with potential therapeutic implications. Additionally, we propose a mechanism that details the role of Wnt11 in fibrosis (Figure 5).

There is ample evidence showing aberrant activation of β-catenin-dependent canonical Wnt signaling in fibrotic lung diseases [19,20,21,22,23]. Previous reports suggest that Wnt3a induces α-SMA expression in mouse fibroblasts, potentially through Smad2, an effect that is reversed by knockdown of β-catenin using siRNA [24]. Selective inhibition of the β-catenin/CBP interaction with the small-molecule inhibitor ICG-001 in mice significantly suppresses β-catenin signaling and attenuates BLM-induced lung fibrosis, as well as renal fibrosis in a ureteral obstruction model [22,25]. Our previous work indicates that Wnt3a/β-catenin-induced α-SMA during myofibroblast differentiation may occur indirectly via activated non-canonical ligands, particularly Wnt11, an effect significantly inhibited by Wnt11 and/or Wnt5a siRNAs [26].

Canonical and non-canonical pathways are not entirely independent, as they can regulate each other. Activation of canonical Wnt3al signaling induces non-canonical Wnt11 and, to a lesser extent, Wnt5a, which in turn inhibits canonical signaling via a negative feedback mechanism [27,28,29]. While Wnt3a can indirectly promote myofibroblast differentiation through non-canonical ligands, this effect is significantly reduced by Wnt11 or Wnt5a siRNAs and completely abolished when both siRNAs are used simultaneously [6,30]. Knocking down β-catenin markedly reduces Wnt11 expression and reverses α-SMA induction in human lung fibroblasts, supporting the idea that Wnt3a-induced myofibroblast differentiation dependents on β-catenin-mediated induction of the non-canonical ligands Wnt11 and likely Wnt5a.

The switch from canonical to non-canonical Wnt pathways has been suggested during cardiac progenitor cell development. Increased canonical Wnt signaling is observed in Wnt11-deficient embryos, whereas treatment with Wnt11 or Wnt5a inhibits expression of β-catenin and its target gene Axin2 in these cardiac progenitors via caspase3 signaling [13,14]. Additionally, Wnt5a attenuates Wnt3a-induced alkaline phosphatase expression in dental follicle cells without affecting β-catenin nuclear translocation [29]. These observations suggest that induced non-canonical signaling provides negative feedback to counteract the initiating canonical signal, thereby blunting its overall effect [6,30]. This mechanism is supported in vivo by the observation that lung canonical ligands, including Wnt3a and Wnt10b, are significantly reduced in BLM-induced pulmonary fibrosis, whereas Wnt11 is the most upregulated gene (over two-fold higher than Wnt5a in our study).

Cross-regulation between the Wnt and TGFβ signaling pathways also contributes to myofibroblast differentiation in fibrosis [7,31,32]. Non-canonical Wnt signaling is activated by TGFβ signaling in lung fibroblasts and is similarly required for TGFβ-induced epithelial–mesenchymal transition (EMT) in renal epithelial cells [6,8]. Both human and mouse lung fibroblasts express constitutive levels of Wnt11, which are lower than Wnt5a, while canonical Wnt3a is undetectable. In contrast, alveolar type II epithelial cells AECIIs express a higher level of Wnt11 relative to fibroblasts, with only limited Wnt3a expression [6]. These differential expression patterns suggest potential crosstalk between ACEIIs and fibroblasts, which was confirmed by co-culture studies of TGFβ-activated AECIIs with fibroblasts. These co-culture studies suggest that AECII-derived Wnt11 can potentially activate myofibroblast differentiation.

Since myofibroblasts are a known source of TGFβ [33], these findings further suggest a positive feedback loop in which AECII–fibroblast interaction sustains myofibroblast induction, thereby promoting fibrosis [6,8]. Further supporting this mechanism, recent studies demonstrate elevated Wnt11 expression in fibrotic lungs alongside the established role of TGFβ in BLM-induced pulmonary fibrosis in mice [34,35,36]. While Wnt11 is required for TGFβ-induced EMT mediated by Smad3 in renal epithelial cells [8], it remained unclear whether Smad3 directly regulates Wnt11 transcription. The present study provides evidence that TGFβ upregulates Wnt11 transcription via direct pSmad3 binding to the Wnt11 promoter. Additionally, the observed binding of β-catenin and TCF4 suggest potential interaction between Smad3 and β-catenin or TCF4, consistent with prior reports of synergistic transcriptional regulation of target genes by Smad3 and β-catenin/LEF1 complexes [32,37]. Our findings provide mechanistic insights into Wnt11 regulation, and future studies incorporating targeted mutagenesis are warranted to confirm and clarify the precise contributions of these binding sites to Wnt11 transcriptional regulation.

A potential limitation of the present study is that the potential off-target Cre activity in the Col1a2-CreERT2 Wnt11 conditional knockout mice was not directly quantified, although this same Cre mouse line has been validated for fibroblast-specific KO in a separate study [38] from our laboratory. We cannot formally exclude variability in non-fibroblast lineages, if there are any. Nevertheless, in this study, Col-Cre-LoxP recombination resulted in a ~77% reduction of Wnt11 expression in collagen-expressing fibroblasts. The concordance between our in vivo phenotype and fibroblast-intrinsic in vitro findings supports a primary role for fibroblast-derived Wnt11 in regulating lung fibrosis in this bleomycin model of lung fibrosis.

## 4. Methods

### 4.1. Mice and BLM Model of Pulmonary Fibrosis

Six-week-old female C57BL/6, Wnt11^fl/fl^ (B6;129-Wnt11tm.1Ser/J, Stock# 030051) and Col-Cre^+/−^ (C57BL/6J-Tg[Col1α2-Cre-ER(T)], stock# 029567) mice were purchased from The Jackson Laboratory (Bar Harbor, ME, USA). Wnt11^fl/fl^ mice were bred with Col1a2-ERT2 Cre^+/−^ mice as before [38]. The resultant Wnt11^fl/fl^,Col1a2-ERT2 Cre^+/−^ mice were used as conditional KOs (Wnt11-ColCre cKO), and their littermates (Wnt11^fl/fl^) were used as controls (referred to as WT). Both WT and cKO mice received tamoxifen (2 mg/mouse, i.p.) for 7 consecutive days to induce Wnt11 deficiency. Off-target activity is not detected previously using the same Col-Cre^+/−^ mouse line [38]. To induce pulmonary fibrosis, bleomycin (BLM, Blenoxane, Princeton, NJ, USA) was dissolved in sterile PBS and instilled by oropharyngeal aspiration at a dose of 2 U/kg body weight (5 mice/group). All animals were housed in a standard vivarium under controlled environmental conditions, with food and water provided ad libitum. This study examined only female animals.

### 4.2. Cell Isolations and Treatments

De-identified primary human lung fibroblasts (HLFs) isolated from control subjects or patients with idiopathic pulmonary fibrosis (IPF) were kindly provided by Dr. Craig A. Henke (University of Minnesota, Minneapolis, MN, USA) and maintained in DMEM supplemented with 10% fetal bovine serum (Sigma-Aldrich, St. Louis, MO, USA). Primary mouse lung fibroblasts (MLFs) were isolated from naïve C57BL/6 mice using a digestion cocktail containing collagenase III and DNase I (Worthington Biochemical Crop., Lakewood, NJ, USA) and maintained in DMEM supplemented with 10% fetal bovine plasma-derived serum (Animal Technologies, Tyler, TX, USA) with the addition of EGF and PDGF (R&D Systems, Inc., Minneapolis, MN, USA) as before [39]. Primary alveolar type II epithelial cells (AECIIs) were isolated using Dispase II (Roche Diagnostics, Indianapolis, IN, USA), and CD16/32 and CD45 expressing cells were negatively selected by magnetic-activated cell sorting separation system (Miltenyi Biotec Inc., San Diego, CA, USA) as before [40]. The AECIIs were then plated and cultured for 2 days on plates coated with 200 μg/mL Matrigel (BD Biosciences, Franklin Lakes, NJ, USA) before use. For Figure 3D, AECIIs were cultured in laminin-coated 2D culture system for differentiation analysis. These cultures progress to an AECII transitional state that does not lead to AECI differentiation and recapitulate the transitional AECII state seen in idiopathic pulmonary fibrosis [16]. Where indicated, the A549 cell line (ATCC, Manassas, VA, USA), primary fibroblasts, or mouse AECIIs were treated with TGFβ (5 ng/mL for human cells and 10 ng/mL for mouse cells) (R&D Systems, Inc., Minneapolis, MN, USA), 5 µM CHIR99021 (Sigma-Aldrich, Burlington, MA, USA) and Wnt3a (200 ng/mL) (R&D Systems, Inc.) for the indicated times. In co-culture experiments, mouse primary AECIIs or human A549 cells were first transfected with Wnt11 siRNAs (Ambion, Austin, TX, USA) for 24 h and then treated with TGFβ for another 48 h. Mouse or human fibroblasts were plated in the bottom chamber of 12-well Transwell plates one day prior to coculture. Primary mouse AECII or A549 cells were placed onto 0.4 µm insert membranes. The coculture cells were incubated for an additional 48 h.

ihAEC2s were kindly provided by Dr. Rachel Zemans and were previously generated and induced as described in previous studies [17,41]. To maintain and expand ihAEC2s in organoids, PSC-derived SFTPC+ alveolar cells (ihAEC2s) were cultured using a 3D Matrigel (Corning product # 356231, Corning Compony, Corning, NY, USA) approach. Briefly, ihAEC2s (1.2 × 10^4^) were mixed with 30 µL of thawed, undiluted Matrigel on ice. This mixture (1.2 × 10^4^ ihAEC2s/30 µL Matrigel/well) was plated as droplets in 24-well plates and maintained in the inducing medium of DMEM containing KGF, Dexamethasone, 8-Br-cAMP and IBMX (3-isobutyl-1-methylxanthine). The cells were passaged every 12–14 days. For the coculture experiment, the ihAEC2-Matrigel droplets were plated onto the insert membranes of 12-well Transwell plates. Wnt11 siRNA transfection using Lipofectamine 3000 (Thermo Fisher Scientific (Waltham, MA, USA) was performed on day 8 for 24 h. This was followed by TGFβ treatment (5 ng/mL) on day 9 for an additional 48 h. After removing the TGFβ by replacing the medium, the ihAEC2s were cocultured with HLFs that had been plated on the bottom chambers 24 h earlier. The cocultured HLFs were harvested 48 h later for analysis.

### 4.3. siRNA Transfection and Adenoviral Transduction

The pre-designed Silencer^®^ select siRNAs for Wnt11 ( s76069 (mouse), s14892 (human) were purchased from Ambion, β-catenin (s436) or control siRNA were purchased from Thermo Fisher Scientific (Waltham, MA, USA). The siRNAs (50 nM) were transfected into the fibroblasts using Lipofectamine™ RNAiMAX (Thermo Fisher Scientific) according to the manufacturer’s protocol.

Adenoviral Cre or control (provided by the University of Michigan Vector Core, Ann Arbor, MI, USA) were transduced into primarily cultured MLFs at 200 MOI for 5 days, followed by treatment with Wnt3a (200 ng/mL) or TGFβ (10 ng/mL) for an additional 24 or 48 h.

### 4.4. Real Time PCR and Western Blotting Analysis

The primers and Taqman probes of the following genes were purchased from Thermo Fisher Scientific: human or mouse α-SMA, type I procollagen, Wnt3a, Wnt5a, Wnt10b, Wnt11, Axin2, lrp5 and Fzd7, 18S. For each qPCR assay, 50 ng of total RNA was used. 18s rRNA was used as an internal control. One-step RT-PCR was performed on a GeneAmp 7500 sequence detection system (Applied Biosystems, Foster City, CA, USA). The results were expressed as 2^−ΔΔCT^. The antibodies used in Western blotting were anti-mouse anti-collagen I (Cat# GTX112731, GenTex, Irvine, CA, USA), anti- mouse α-SMA(catalog number A2547; Sigma-Aldrich, Burlington, MA, USA) and anti-GAPDH (G9295, Sigma-Aldrich) was used as a loading control.

### 4.5. Chromatin Immunoprecipitation (ChIP) Assay

This was undertaken to analyze for binding of β-catenin, TCF4 or pSmad3 to the human α-SMA or Wnt11 promoter region using a ChIP assay kit (Millipore, Temecula, CA, USA), according to the manufacturer’s protocol. Briefly, HLFs (2 × 10^6^) were treated with 1% formaldehyde for DNA–protein crosslinking after treatments. The cells were lysed with SDS lysis buffer followed by DNA shearing by sonication. The 1% lysis buffer was saved for input control. The lysates were incubated with anti-non-phospho (active) β-catenin antibodies (S33/S37, clone D13A1, Cat# 8814), TCF4/TCF7L2 (clone C48H11, Cat# 2565S) or p-Smad3 (S423/425, clone C25A9, Cat# 9520S, Cell Signaling Technology, Danvers, MA, USA), respectively. The purified DNA fragments were subjected to real-time PCR using SYBR Select Master Mix (Applied Biosystems) and performed on a GeneAmp 7500 sequence detection system. The sequences of the PCR primers for the human α-SMA or Wnt11 promoter regions are as follows: ChIP primers for β-catenin, TCF4 and Smad3 bindings on human α-SMA promoter (−725 to −831), For P: 5′ GCAGAGAGGAGGGCTACAGA-3′; Rev P: 5′-TAGGGACCATCCTTTGACCA-3′.

ChIP primers for Smad3 binding on human Wnt11 promoter: Smad3 ChIP primer1 (−882 to −827).

For P: 5′-GAGCAGATGCGATCGTGTTA-3′; Rev P: 5′-AAGAGCGAAACTCCGTCTCA-3′;

Smad3 ChIP primer2 (−601 to −459), For P: 5′-CTCGACCTCCCAAAGTGCT-3′; Rev P: 5′-GGCAATTCTCCTTCCCAAAC-3′; Smad3 ChIP primer3 (−385 to −262), For P: 5′-CCTGGACTCCTGATTCCTCA-3′; Rev P: 5′-GACACAGCGAGAGGGAGAAG-3′.

### 4.6. Promoter Activity Assay

Four serially truncated human Wnt11 promoter–luciferase constructs (from −997, −500, −300 and −53 from TSS to +74, Figure 4A) were generous gifts from Dr. Soon Young Shin (Sanghuh College of Lifesciences, Konkuk University, Seoul, Republic of Korea) [42]. Each construct (250 ng) was co-transfected with 25 ng *Renilla* luciferase control vector (pRL-SV40, Promega Corporation, Madison, WI, USA) into HLFs plated in 24-well plates using Lipofectamine 3000 (Thermo Fisher Scientific). After 24 h, the cells were treated using CHIR99021, Wnt3a or TGFβ and harvested at the indicated time points. The activity of firefly or *Renilla* luciferase was measured using the dual-luciferase reporter assay system (Promega) with a Veritas Microplate Luminometer (Turner Biosystems, Sunnyvale, CA, USA). The relative luciferase activity was calculated by normalizing firefly luciferase activity to that of *Renilla* luciferase. Experiments with each construct were repeated at least 3 times, and relative light units were expressed as means ± SD.

### 4.7. Histological Staining and Ashcroft Scoring

The lungs were perfused through the heart to remove residual red blood cells, inflated intratracheally and subsequently fixed by perfusion with 10% buffered formalin for 24 h. Lung tissue was then paraffin-embedded, sectioned and stained with hematoxylin and eosin (H&E). Additional sections were stained with Masson’s trichrome and evaluated for fibrosis using the Ashcroft scoring system. Blinded observers assigned fibrosis scores ranging from 0 (normal lung) to 8 (severe architectural distortion with complete fibrotic obliteration) according to established Ashcroft criteria [43]. For each lung tissue section, fifteen randomly selected fields were scored, and the average value was used for analysis.

### 4.8. Statistics

All data were expressed as mean ± S.D. unless otherwise indicated. Differences between the means of various treatment and control groups were assessed for statistical significance by one-way ANOVA with Tukey’s multiple comparisons using GraphPad Prism (version 9.5.1, GraphPad Software, San Diego, CA, USA). Student’s *t*-test was used for comparisons of 2 groups. A *p* value less than 0.05 was considered significant. * *p* < 0.05, ** *p* < 0.01, *** *p* < 0.005, **** *p* < 0.0001 in all figures. No animals (or data points) were excluded from the statistical analyses.

### 4.9. Study Approval

All animal studies were reviewed and approved by the Institutional Animal Care and Use Committee at the University of Michigan.

## 5. Conclusions

Our data highlight the functional importance of Wnt11 in promoting myofibroblast differentiation. Consistent with this, depletion of Wnt11 in fibroblasts ameliorated fibrosis in vivo. In addition, Wnt11 appears to mediate crosstalk between AECII and fibroblasts in a paracrine manner, potentially enhancing fibrotic progression. Finally, TGFβ may upregulate Wnt11 at the transcriptional level, perhaps via a direct Smad3 binding to the α-SMA promoter. Together, these findings indicate coordinated crosstalk of non-canonical Wnt with the canonical as well as TGFβ/Smad3 pathways in driving myofibroblast differentiation and fibrosis. While our results provide important mechanistic insights, future studies will be necessary to further delineate the precise interactions among these pathways and their relative contributions to fibrotic progression.

## Data Availability

The raw data supporting the conclusions of this article will be made available by the authors upon request.
